# Small airway disease in post-acute COVID-19 syndrome, a non-conventional approach in three years follow-up of a patient with long COVID: a case report

**DOI:** 10.1186/s13256-023-04113-7

**Published:** 2023-09-11

**Authors:** Ivan Cherrez-Ojeda, Maria F. Osorio, Karla Robles-Velasco, Juan C. Calderón, Arturo Cortés-Télles, Jorge Zambrano, Cristian Guarderas, Belen Intriago, Laura Gochicoa-Rangel

**Affiliations:** 1 Universidad Espíritu Santo, Km. 2.5 Vía La Puntilla, Samborondón, 0901-952 Ecuador; 2 Respiralab, Respiralab Research Group, Guayaquil, Ecuador; 3 Departamento de Neumología y Cirugía de Tórax, Hospital Regional de Alta Especialidad de Yucatán, Mérida, Mexico; 4 Centro de enfermedades respiratorias, rehabilitación y sueño (CERS), Guayaquil, Ecuador; 5 Departamento de Fisiología Respiratoria, Instituto Nacional de Enfermedades Respiratorias “Ismael Cosío Villegas”, Ciudad de México, Mexico

**Keywords:** COVID-19, Lung Clearance Index, Multiple breathing washout, Oscillometry, Small airway disease

## Abstract

**Background:**

Small airways disease (SAD), a novel finding described in post-acute COVID-19 patients, should be suspected when respiratory symptoms continue, air trapping persists on expiratory CT scans, and imaging findings fail to improve despite objectively better conventional pulmonary function test (PFT) parameters. The forced oscillation technique (FOT) and Multiple breathing washout (MBW) are both very sensitive methods for detecting anomalies in the peripheral airways.

**Case presentation:**

We discuss the case of a 60-year-old Hispanic patient who had severe COVID-19 pneumonia and developed dyspnea, fatigue, and limited daily activity a year later. The PFTs revealed restrictive lung disease, as seen by significant diffusing capacity of the lungs for carbon monoxide (DLCO) decrease, severe desaturation, and poor 6-min walk test (6MWT) performance. The patient was treated with lowering corticosteroids as well as pulmonary rehabilitation (PR). During the 24-month follow-up, the dyspnea and fatigue persisted. On PFTs, 6MWT performance and restricted pattern improved slightly, but MBW discovered significant ventilatory inhomogeneity. FOT revealed substantial peripheral airway obstructive abnormalities. On CT scans, air trapping and ground-glass opacities (GGO) improved somewhat. The patient used a bronchodilator twice a day and low-dose inhaled corticosteroids (160 µg of budesonide and 4.5 µg of formoterol fumarate dihydrate) for nine months. PR sessions were resuming. The restricting parameters were stabilized and the DLCO had normalized after 36 months, with a 6MWT performance of 87% but significant desaturation. The CT scan revealed traction bronchiectasis, low GGO, and persistent air trapping. Without normalization, FOT and MBW scores improved, indicating small airway disease.

**Conclusions:**

The necessity of integrating these tests when detecting SAD is emphasized in our paper. This article lays the foundation for future research into the best ways to manage and monitor SAD in post-acute COVID-19 patients.

## Background

Post-acute COVID-19 syndrome (PACS) afflicts more than half of patients two years after the acute phase, placing a significant burden on individuals and the healthcare system [1]. Conventional pulmonary function tests (PFTs) and imaging studies, such as computed tomography (CT) scans, are used to follow up post-acute COVID-19 patients [2]. However, small airway disease (SAD), as a potential cause of persistent clinical symptoms and imaging abnormalities, has received little attention [3]. SAD is the inflammation of peripheral bronchioles with ≤ 2 mm internal diameter due to direct bronchiole or vascular lesions, or by immune responses associated with interstitial and bronchial alterations [4]. Non-conventional PFTs, such as the forced oscillation technique (FOT) and multiple breathing washout (MBW) [5], can detect SAD and complement conventional tests. We present a case of persistent respiratory symptoms assessed by CT scan, FOT, and MBW over a period of three years, attributable to the SAD findings.

## Case presentation

**Fig. 1:**
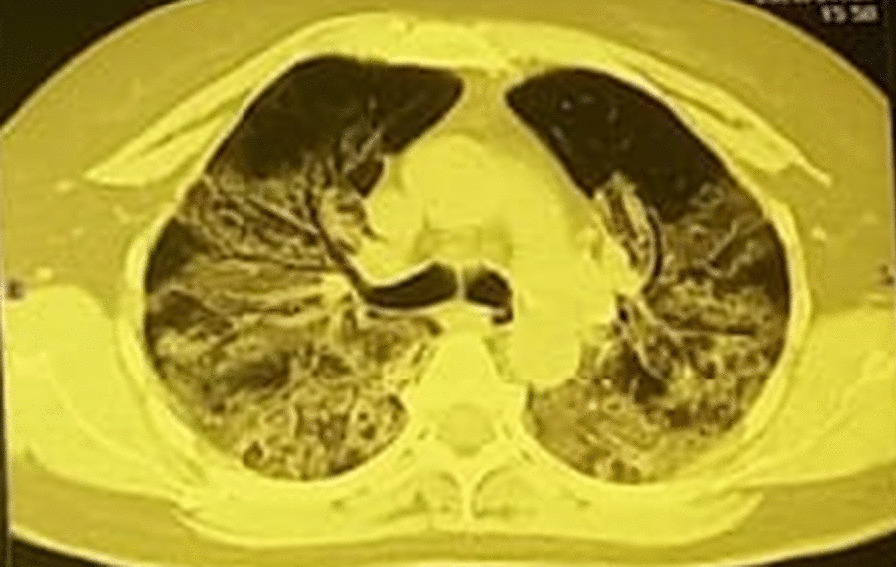
Initial computed tomography (CT) scan showing bilateral consolidations and ground-glass opacities involving most of the lung parenchyma

**Fig. 2:**
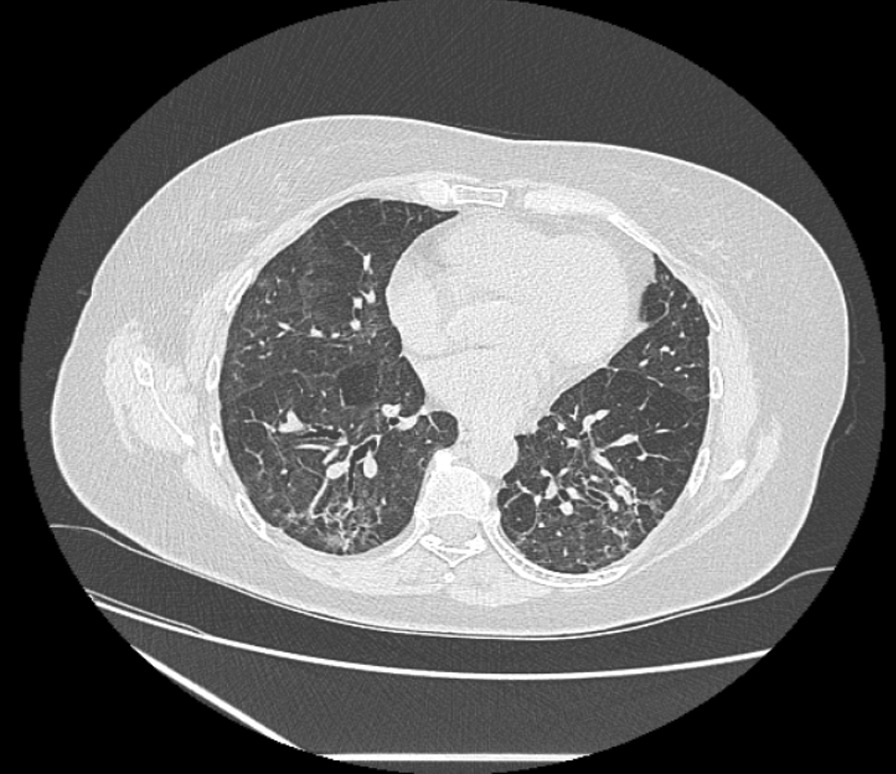
12-month follow-up computed tomography scan showing mild, diffuse, bilateral ground-glass opacities associated with bronchiectasis in the upper and lower lobes of the lungs. Air trapping area in the posterior segments of the upper lobes and in the basal segments of the anterior lower lobes, evident in the expiratory phase

In April 2020, a 60-year-old Hispanic patient developed severe COVID-19 pneumonia that was treated at home because the healthcare system was oversaturated. The initial CT scan revealed bilateral ground-glass opacities (GGO) and lung parenchymal consolidations (Fig. 1). A year after the acute infection, the patient had persistent dyspnea, fatigue, and a limited ability to resume daily activities. The PFTs revealed restrictive lung disease with a moderate decrease in DLCO, significant desaturation, and poor performance on the 6-min walk test (Table 1). CT scans of the chest revealed air trapping during the expiratory phase as well as structural lesions in the parenchyma: bilateral diffuse GGO associated with bronchiectasis and interlobular septal thickening (Fig. 2). The patient was given corticosteroids, which were gradually tapered off, oxygen via a nasal cannula, and pulmonary rehabilitation (PR).

**Table 1:** Pulmonary function tests during 36 months follow-up

Pulmonary function tests	Months of follow-up post-acute COVID-19 infection								
	3	6	10	12	16	22	24	26	36
Spirometry									
FVC [L]									
Absolute value	1.06^a^	1.38^a^	1.57^a^	1.43^a^	1.63^a^	–	1.65^a^	–	1.64^a^
Predicted (%)	39^a^	51^a^	59^a^	53^a^	62^a^	–	62^a^	–	62^a^
FEV1 [L]									
Absolute value	0.92^a^	1.13^a^	1.24^a^	1.14^a^	1.30^a^	–	1.35^a^	–	1.32^a^
Predicted (%)	44^a^	54^a^	60^a^	55^a^	63^a^	–	66^a^	–	65^a^
FEV1/FVC [%]									
Absolute value	87	81.7	79.5	79.9	79.9	–	82	–	80.5
Predicted (%)	113	106	101	102	102	–	105	–	103
DLCO									
DLCO [ml/min/mmHg]									
Absolute value	8.3^a^	11.7^a^	10.3^a^	10.6^a^	11.3^a^	–	17.1	–	15.2
Predicted (%)	41^a^	58^a^	51^a^	52^a^	56^a^	–	85	–	76
DLCO/VA (KCO) [ml/min/mmHg]									
Absolute value	4.52	4.32	4.24	4.84	4.73	–	4.45	–	4.87
Predicted (%)	85	82	80	91	89	–	84	–	92
TLC sb [L]									
Absolute value	2	2.86	2.59^a^	2.34^a^	2.55^a^	–	4	–	3.26
Predicted (%)	51	72	65	59	65	–	101	–	83
6MWT									
6-MWD (m)	264	359	390	–	390	–	339	–	405
Performance (%)	55	75	81	–	85	–	72	–	87
Basal SpO_2_ (%)	93	97	97	–	95	–	97	–	98
Maximum desaturation	82	92	92	–	95	–	91	–	94
MBW									
FRC mb [L]									
Absolute value	–	–	–	–	–	–	1.01^a^	1.21^a^	1.31^a^
Predicted (%)	–	–	–	–	–	–	49^a^	58^a^	63^a^
LCI									
Absolute value	–	–	–	–	–	–	11.86^a^	13.57^a^	11.55^a^
Predicted (%)	–	–	–	–	–	–	178^a^	203^a^	173^a^
FOT									
Rrs5 [cmH_2_O/(L/s)]									
insp Z score	–	–	–	–	–	1.91^a^	1.42	0.96	1.51
exp Z score	–	–	–	–	–	2.09^a^	1.14	1.45	1.4
tot Z score	–	–	–	–	–	2.01^a^	1.25	1.26	1.45
Xrs5 [cmH_2_O/(L/s)]									
insp Z score	–	–	–	–	–	− 3.09^a^	− 3.01^a^	− 2.31^a^	32.64^a^
exp Z score	–	–	–	–	–	− 2.3^a^	− 2.19^a^	− 0.74	− 2.25^a^
tot Z score	–	–	–	–	–	− 2.67^a^	− 2.54^a^	− 1.37	− 2.38^a^
Rrs11 [cmH_2_O/(L/s)]									
insp Z score	–	–	–	–	–	1.15	0.41	0.03	–
exp Z score	–	–	–	–	–	1.98^a^	1.02	1.27	1
tot Z score	–	–	–	–	–	1.65^a^	0.78	0.85	1.3
Xrs11 [cmH2O/(L/s)]									
insp Z score	–	–	–	–	–	− 3.05^a^	− 2.49^a^	− 2.19^a^	− 2.42^a^
exp Z score	–	–	–	–	–	− 2.72^a^	− 2.59^a^	− 2.23^a^	− 2.5^a^
tot Z score	–	–	–	–	–	− 2.88^a^	− 2.55^a^	− 2.22^a^	− 2.5^a^
Rrs19 [cmH_2_O/(L/s)]									
insp Z score	–	–	–	–	–	0.92	− 0.04	− 0.19	0.73
exp Z score	–	–	–	–	–	1.72^a^	0.71	1.27	1.03
tot Z score	–	–	–	–	–	1.4	0.42	0.78	0.92
ΔXrs (M)	–	–	–	–	–	− 0.48	− 0.49	− 0.87	− 0.23
R5-R19 insp (M)	–	–	–	–	–	2.09	2.28	1.71	–
Cardiopulmonary exercise testing									
Peak *V*O_2_, mL/min/kg	–	–	–	–	–	–	–	–	16.2^a^
Peak *V*O_2_, % predicted	–	–	–	–	–	–	–	–	56^a^
AT, mL/min/kg	–	–	–	–	–	–	–	–	14.5
AT, % predicted	–	–	–	–	–	–	–	–	54.5
Breathing reserve, %	–	–	–	–	–	–	–	–	19.3
HRR, %	–	–	–	–	–	–	–	–	< 1
Peak heart rate, beats/min	–	–	–	–	–	–	–	–	6.4^a^
Peak HR, % predicted	–	–	–	–	–	–	–	–	57.4^a^
Peak O_2_ pulse, mL/beat	–	–	–	–	–	–	–	–	35.7^a^
Peak O_2_ pulse, % predicted	–	–	–	–	–	–	–	–	80.69^a^
VE/VCO_2_ slope	–	–	–	–	–	–	–	–	162^a^
VE/MVV, %	–	–	–	–	–	–	–	–	103.18^a^
Peak HR, % predicted	–	–	–	–	–	–	–	–	57.4^a^
Peak O_2_ pulse, mL/beat	–	–	–	–	–	–	–	–	35.7^a^
Peak O_2_ pulse, % predicted	–	–	–	–	–	–	–	–	80.69^a^
VE/VCO_2_ slope	–	–	–	–	–	–	–	–	162^a^
VE/MVV, %	–	–	–	–	–	–	–	–	103.18^a^

^a^Indicates value outside the normal range

*FVC* forced vital capacity, *FEV1* forced expiratory volume in 1 s, *DLCO* diffusing capacity of carbon monoxide, *VA* alveolar ventilation, *DLCO/VA (KCO)* carbon monoxide transfer coefficient, *TLC* total lung capacity, *6-MWT* 6-min walking test, *6-MWD* 6-min walking distance, *SpO2* peripheral oxygen saturation, *FOT* forced oscillation technique, *Rrs5* resistance of respiratory system at 5 Hz, *Xrs5* reactance of respiratory system at 5 Hz, *Rrs11* resistance of respiratory system at 11 Hz, *Xrs11* reactance of respiratory system at 11 Hz, *Rrs19* resistance of respiratory system at 19 Hz, *MBW* multiple breath washout, *FRC* functional residual capacity, *LCI* lung clearance index, *VO*_*2*_ oxygen consumption, *AT* anaerobic threshold, *HRR* heart rate reserve, *HR* heart rate, *VE/VCO*_*2*_ ventilatory efficiency, *VE/MVV* ventilatory reserve

**Fig. 3:**
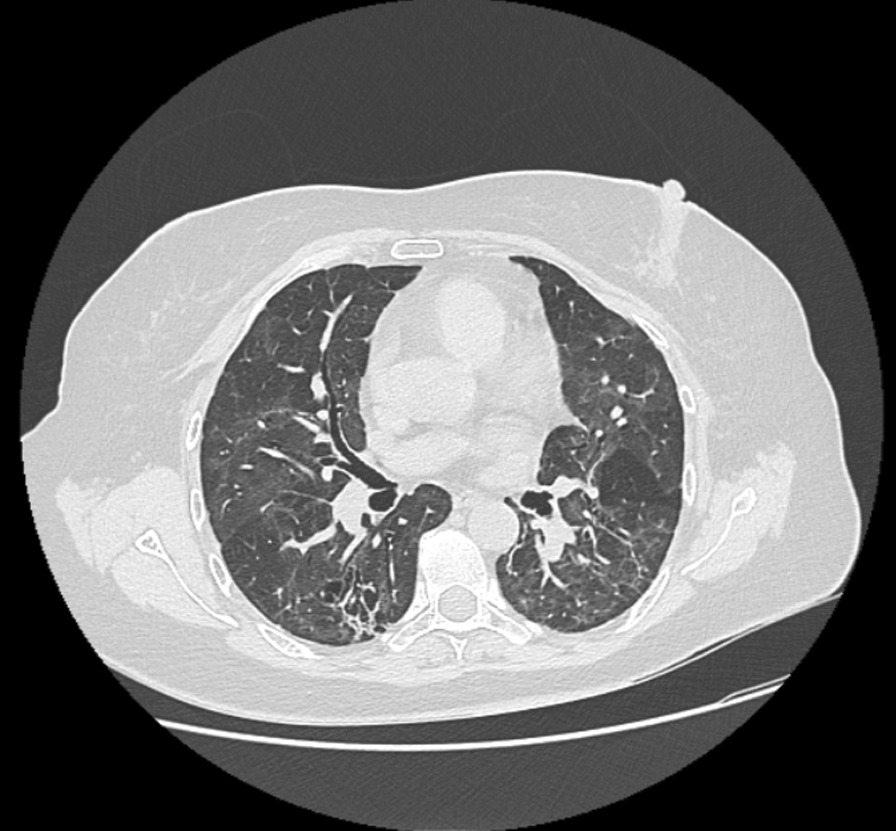
24-month computed tomography scan follow-up with mild, diffuse, bilateral ground-glass opacities associated with septal thickening interlobular as well as traction bronchiectasis in the lower lobes with a right predominance and images dense subpleural lines, compatible with areas of pneumonitis associated with pulmonary interstitial changes, with images of bilateral air trapping

At the 24th-month follow-up, the patient still experienced dyspnea and exhaustion during exercise. pulmonary function tests revealed a modest improvement in the 6-min walk test performance percentage and restrictive pattern (Table 1). Forced oscillation technique revealed severe obstructive changes in the peripheral airways, which were consistent with multiple breathing washout findings of significant ventilatory inhomogeneity. Computed tomography scans showed a mild improvement in air trapping and ground-glass opacities (Fig. 3). The patient began taking a bronchodilator twice daily for nine months, along with low-dose inhaled corticosteroids (160 µg of budesonide and 4.5 µg of formoterol fumarate dihydrate). The PR sessions were also reinstated.

**Fig. 4:**
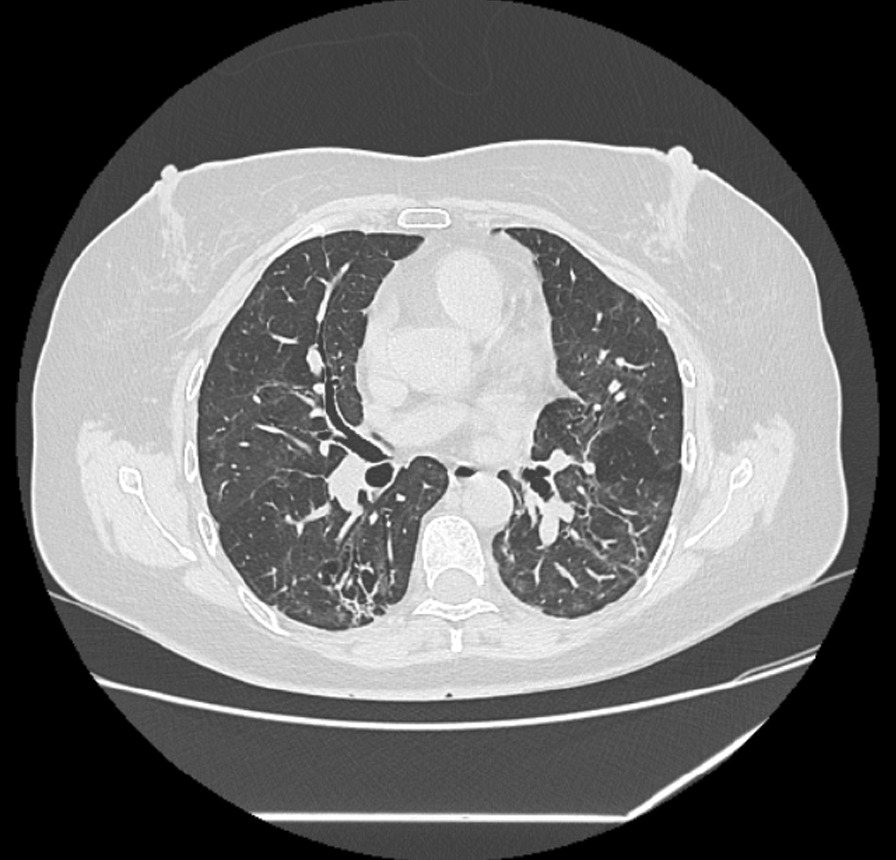
36-month CT scan follow-up, GGOs of lower density than in a previous study, air trapping in the expiratory phases, thickening of bilateral interlobular septa, traction bronchiectasis

After 36 months, the restrictive parameters remained stable and the DLCO remained within normal ranges, with a performance of 87% in the 6MWT but still having significant desaturation (Table 1). The CT scan revealed that the traction bronchiectasis had persisted, with reduced GGO and air trapping (Fig. 4). Furthermore, FOT and MBW measurements showed an improvement in scores without normalization, classifying the patient as having small airway disease. As a result of these findings, we decided to perform a Cardiopulmonary Exercise Test (CPET), which resulted in a significantly lower VO2peak (16.2 ml/min/kg, 56% predicted), a low peak oxygen pulse, inefficient ventilation, and an elevated maximum heart rate (Table 1). The patient was referred to the cardiology service for further workup.

## Discussion and conclusions

Post-acute COVID-19 syndrome (PACS) is present in more than half of patients, even after 2 years of acute disease [1, 3, 6]. Fatigue and dyspnea are the most common manifestations [1, 7]. Current literature has described the most common effects of SARS-CoV-2 on lung structure and function as altered diffusion capacity and restrictive and obstructive patterns [6]; however, little is known about the role of SAD.

Our patient's CT showed persistent findings of GGOs and air trapping. More than 50% of CT scans of post-acute COVID-19 patients reported air trapping [8, 9], which has been correlated with more severe long-term outcomes despite the severity of the acute infection [10]. Air trapping is explained by capillary wall injury and small airway wall thickening as partial or complete airway obstruction in regions of the lung induced by pro-inflammatory factors [3]. Air trapping on a chest CT scan can serve as a biomarker of functional small airway disease [3], which is challenging to assess through traditional PFTs. This is because PFTs tend to remain normal until the disease has advanced to a stage where 2/3 of the bronchioles are already obstructed [10]. The GGOs occur as a result of persistent damage in the interstitium due to the ongoing residual inflammatory process by neutrophil extracellular traps (NETs) that persist even when the virus has cleared [11]. Both air trapping and GGOs can lead to a ventilation/perfusion mismatch, reducing oxygenation [8].

Patients presenting with air trapping and no airflow obstruction by spirometry, as observed in our patient, suggest the involvement of small rather than large airways [3, 10], which can be caused by mucus impaction, a primary abnormality of the pulmonary microvasculature (endotheliitis and microthrombosis) or constrictive bronchiolitis that produce a ventilation/perfusion mismatch [8]. It can be detected by two methods: oscillometry and MBW through its lung clearance index (LCI) and Functional Residual Capacity (FRC) [5]. Both have shown advantages over spirometry as a way to monitor "silent" airway remodeling, being useful tools for tracking the progression of early structural airway disease that are currently undetected with spirometry [12, 13]. Oscillometry has a high sensitivity for peripheral lung dysfunction, it is easy to perform in adults and non-cooperative patients and can identify lung disease earlier than conventional PFT [14].

Santus et al. [15] presented lung function tests based on complexity, which included FOT, Fractional Exhaled Nitric Oxide (FeNO), MBW, which are easily performed on adults and non-cooperative patients, allowing for earlier detection of SAD [3, 6, 16]. In the case of our patient, FOT and MBW results showed evidence of SAD, and FENO was within normal limits. These results may explain her exercise limitations, despite the normalization of PFT parameters [16, 17]. A study found abnormal oscillometry results in 88.1% of participants at 2 months and 71.2% at 5 months, highlighting its effectiveness in detecting lung disease and monitoring its progression [6].

Following the recognition of SAD, the question of what therapy may be offered to the patient arises. There is no consensus about the management of PACS with lung damage, and it varies according to the patient's evolution. Bronchodilator therapy should be included in the treatment plan since it can enhance functional outcomes [18]; however, how long it should be used is unknown. A study found that the use of inhaled long-acting bronchodilators or oral and/or inhaled corticosteroids three months after COVID-19 resulted in improved quality of life 15 months later [17]. In the present case, a long-acting beta 2-agonist (LABAs) and inhaled corticosteroid for 9 months were initiated, showing an improvement in oscillometric and MBW measurements after a year of follow-up.

Although there is current interest in SAD as a cause of symptom persistence in post-COVID patients, little is known about the appropriate therapeutics for this subset of patients. Maximizing drug deposition at the site of disease is crucial, and selecting the appropriate drug molecule and inhaler device plays a vital role in achieving this goal [15, 19]. Further research is needed to determine if smaller particle aerosols should be employed as extra fine inhaled formulations to target distal airways in post-COVID individuals with symptoms of SAD and for how long.

It is unclear to what extent pulmonary sequelae may persist, and more studies need to be done. There is no consensus about the diagnostic and therapeutic approach in patients with persisting exercise limitations and dyspnea after COVID-19. It is important to note that a multidisciplinary approach that includes small airway assessment using FOT and MBW might help identify the causes and address the therapeutic interventions. Our report paves the way for further longitudinal studies to determine whether these findings improve over time or whether they lead to persistent or progressive lung disease.

## Data Availability

The datasets used and/or analyzed during the current study are available from the corresponding author upon reasonable request.

## Abbreviations

SAD: Small airway disease
PFTs: Pulmonary function tests
FOT: Forced oscillation technique
MBW: Multiple breathing washout
PR: Pulmonary rehabilitation
GGO: Ground-glass opacities
CPET: Cardiopulmonary exercise test

## References

[1] Fernández-de-las-Peñas C, Rodríguez-Jiménez J, Cancela-Cilleruelo I, Guerrero-Peral A, Martín-Guerrero JD, García-Azorín D (2022). Post-COVID-19 symptoms 2 years after SARS-CoV-2 infection among hospitalized vs nonhospitalized patients. JAMA Netw Open..

[2] Cherrez-Ojeda I, Cortés-Telles A, Gochicoa-Rangel L, Camacho-Leon G, Mautong H, Robles-Velasco K (2022). Challenges in the management of post-COVID-19 pulmonary fibrosis for the Latin American population. J Pers Med..

[3] Cho JL, Villacreses R, Nagpal P, Guo J, Pezzulo AA, Thurman AL (2021). Small airways disease is a post-acute sequelae of SARS-CoV-2 infection. medRxiv.

[4] Burgel PR, Bergeron A, de Blic J, Bonniaud P, Bourdin A, Chanez P (2013). Small airways diseases, excluding asthma and COPD: an overview. Eur Respir Rev.

[5] Cherrez-Ojeda I, Robles-Velasco K, Osorio MF, Calderon JC, Bernstein JA (2022). Current needs assessment for using lung clearance index for asthma in clinical practice. Curr Allergy Asthma Rep.

[6] Lopes AJ, Litrento PF, Provenzano BC, Carneiro AS, Monnerat LB, da Cal MS (2021). Small airway dysfunction on impulse oscillometry and pathological signs on lung ultrasound are frequent in post-COVID-19 patients with persistent respiratory symptoms. PLoS ONE.

[7] Cherrez-Ojeda I, Robles-Velasco K, Osorio MF, Cottin V, Vergara Centeno J, Felix M (2021). Follow-up of two cases of suspected interstitial lung disease following severe COVID-19 infection shows persistent changes in imaging and lung function. Clin Case Rep.

[8] Mogami R, Araújo Filho RC, CoboChantong CG, Santosde Almeida FC, Baptista Koifman AC, Jauregui GF (2022). The importance of radiological patterns and small airway disease in long-term follow-up of postacute COVID-19: a preliminary study. Radiol Res Pract..

[9] Franquet T, Giménez A, Ketai L, Mazzini S, Rial A, Pomar V (2022). Air trapping in COVID-19 patients following hospital discharge: retrospective evaluation with paired inspiratory/expiratory thin-section CT. Eur Radiol.

[10] Cho JL, Villacreses R, Nagpal P, Guo J, Pezzulo AA, Thurman AL (2022). Quantitative chest CT assessment of small airways disease in post-acute SARS-CoV-2 infection. Radiology.

[11] Barnes BJ, Adrover JM, Baxter-Stoltzfus A, Borczuk A, Cools-Lartigue J, Crawford JM, et al. Targeting potential drivers of COVID-19: neutrophil extracellular traps. J Exp Med. 2020;217(6). 10.1084/jem.20200652 PMC7161085 32302401

[12] Macleod KA, Horsley AR, Bell NJ, Greening AP, Innes JA, Cunningham S (2008). Ventilation heterogeneity in children with well controlled asthma with normal spirometry indicates residual airways disease. Thorax.

[13] Saglani S, Malmström K, Pelkonen AS, Malmberg LP, Lindahl H, Kajosaari M (2005). Airway remodeling and inflammation in symptomatic infants with reversible airflow obstruction. Am J Respir Crit Care Med.

[14] Kaminsky DA, Simpson SJ, Berger KI, Calverley P, de Melo PL, Dandurand R (2022). Clinical significance and applications of oscillometry. Eur Respir Rev.

[15] Santus P, Radovanovic D, Pecchiari M, Ferrando M, Tursi F, Patella V (2020). The relevance of targeting treatment to small airways in asthma and COPD. Respir Care.

[16] Candemir I, Ergun P, Şahin ME, Karamanli H. Relationship between exercise capacity and impulse oscillometry parameters after COVID-19 infections. Wien Klin Wochenschr. 2022;1–6. 10.1007/s00508-022-02137-5 PMC9802014 36583749

[17] Kooner HK, McIntosh MJ, Matheson AM, Abdelrazek M, Albert MS, Dhaliwal I, et al. Post-acute COVID-19 syndrome: 129Xe MRI ventilation defects and respiratory outcomes one year later. Radiology. 2023;222557. 10.1148/radiol.222557 PMC9926501 36749209

[18] Maniscalco M, Ambrosino P, Fuschillo S, Stufano S, Sanduzzi A, Matera MG (2021). Bronchodilator reversibility testing in post-COVID-19 patients undergoing pulmonary rehabilitation. Respir Med.

[19] Anderson S, Atkins P, Bäckman P, Cipolla D, Clark A, Daviskas E (2022). Inhaled medicines: past, present, and future. Pharmacol Rev.

